# A phase III randomized-controlled study of safety and immunogenicity of DTwP-HepB-IPV-Hib vaccine (HEXASIIL^®^) in infants

**DOI:** 10.1038/s41541-024-00828-w

**Published:** 2024-02-22

**Authors:** Hitt Sharma, Sameer Parekh, Pramod Pujari, Sunil Shewale, Shivani Desai, Anand Kawade, Sanjay Lalwani, M. D. Ravi, Veena Kamath, Jagannath Mahopatra, Ganesh Kulkarni, Deepak Tayade, Padmasani Venkat Ramanan, Kheya Ghosh Uttam, Lalit Rawal, Avinash Gawande, N. Ravi Kumar, Nishikant Tiple, Jayant Vagha, Pareshkumar Thakkar, Prashant Khandgave, Bhaskar Jedhe Deshmukh, Anurag Agarwal, Vikas Dogar, Manish Gautam, K. S. Jaganathan, Rakesh Kumar, Inderjit Sharma, Sunil Gairola

**Affiliations:** 1 Department of Clinical Research and Pharmacovigilance, Serum Institute of India Pvt. Ltd., Pune, India; 2 Department of Pediatrics, KEM Hospital Research Centre, Vadu, Pune, India; 3 Department of Pediatrics, Bharati Vidyapeeth (Deemed to be University) Medical College & Hospital, Pune, India; 4 Department of Pediatrics, JSS Hospital, Mysuru, India; 5 Department of Community Medicine, Kasturba Medical College, Manipal Academy of Higher Education (MAHE), Manipal, India; 6 Department of Pediatrics, Hamdard Institute of Medical Science and Research, New Delhi, India; 7 Department of Pediatrics, Sanjeevani Children’s Hospital, Aurangabad, India; 8 Department of Pediatrics, Mahatma Gandhi Mission’s Medical College and Hospital, Aurangabad, India; 9 Department of Pediatrics, Sri Ramachandra Hospital, Chennai, India; 10 Department of Pediatrics, Institute of Child Health, Kolkata, India; 11 Department of Pediatrics, Grant Medical Foundation Ruby Hall Clinic, Pune, India; 12 Department of Pediatrics, Government Medical College and Hospital, Nagpur, India; 13 Department of Pediatrics, Niloufer Hospital, Hyderabad, India; 14 Department of Pediatrics, Government Medical College, Chandrapur, India; 15 Department of Pediatrics, Acharya Vinoba Bhave Rural Hospital, Wardha, India; 16 Department of Pediatrics, Sir Sayajirao General Hospital, Baroda, India; 17 Department of Pediatrics, Pulse Multispeciality Hospital, Pune, India; 18 Department of Pediatrics, Baramati Hospital, Pune, India; 19 Department of Pediatrics, Maulana Azad Medical College and Lok Nayak Hospital, New Delhi, India; 20 Department of Quality Control, Serum Institute of India Pvt. Ltd, Pune, India; 21 Production Department, Serum Institute of India Pvt. Ltd, Pune, India

**Keywords:** Phase III trials, Vaccines, Randomized controlled trials

## Abstract

A fully liquid hexavalent containing Diphtheria (D), Tetanus (T) toxoids, whole cell Pertussis (wP), Hepatitis B (Hep B), type 1, 2, 3 of inactivated poliovirus (IPV) and *Haemophilus influenzae* type b (Hib) conjugate vaccine (DTwP-HepB-IPV-Hib vaccine, HEXASIIL^®^) was tested for lot-to-lot consistency and non-inferiority against licensed DTwP-HepB-Hib + IPV in an open label, randomized Phase II/III study. In Phase III part, healthy infants received DTwP-HepB-IPV-Hib or DTwP-HepB-Hib + IPV vaccines at 6, 10 and 14 weeks of age. Blood samples were collected prior to the first dose and 28 days, post dose 3. Non inferiority versus DTwP-HepB-Hib + IPV was demonstrated with 95% CIs for the treatment difference for seroprotection/seroconversion rates. For DTwP-HepB-IPV-Hib lots, limits of 95% CI for post-vaccination geometric mean concentration ratios were within equivalence limits (0.5 and 2). Vaccine was well-tolerated and no safety concerns observed.

**Clinical Trial Registration** – CTRI/2019/11/022052

## Introduction

Tetanus (T), diphtheria (D), pertussis (whopping cough), hepatitis B (Hep B), poliomyelitis, and invasive diseases caused by *Haemophilus influenzae* type b (Hib) are serious childhood infectious diseases. Immunization against these six diseases has been recommended by the World Health Organization (WHO) and for this, expanded program on immunization (EPI) initiated by WHO, are well implemented in most countries worldwide^1^. Since its inception, many different DT based combination vaccines were introduced in immunization programs^2^. Combination vaccines have been formulated and introduced to minimize the complexity of vaccine supply, logistics, and vaccination execution. The use of combined vaccines simplifies vaccination programs while reducing implementation costs and is considered as a valuable approach in improving immunization coverage^3–5^. Also, vaccines combining Inactivated Poliomyelitis vaccine (IPV) are of great public health importance in the view of WHO’s Strategic Advisory Group of Experts recommendation to introduce IPV in the immunization programs^6^.

Whole cell pertussis (wP) based pentavalent DTwP-HepB-Hib vaccine (Pentavac^**®**^ SD) and IPV (Poliovac^**®**^) vaccine manufactured by Serum Institute of India Pvt. Ltd. (SIIPL) are WHO pre-qualified and being supplied in large quantities to many countries in Africa, Asia, Latin America and Eastern Europe over the last decade^7^. Now, SIIPL has indigenously developed and manufactured a ready-to-use, fully liquid hexavalent DTwP-HepB-IPV-Hib vaccine (HEXASIIL^**®**^) including established DTwP-HepB-Hib and IPV antigens. Ready-to-use vaccine decreases the time needed for vaccine preparation, administration and reduce multiple injections thus improving vaccine compliance helping to increase vaccination coverage. Safety and immunogenicity of this new vaccine was assessed in toddlers aged 16–24 months in a Phase I clinical study. The vaccine induced robust immune response and no safety concerns were observed^8^. The present Phase III study was conducted in infants following primary vaccination to demonstrate safety and immunogenic non-inferiority of DTwP-HepB-IPV-Hib vaccine to the licensed DTwP-HepB-Hib (Pentavac^**®**^SD) and IPV (Poliovac^**®**^) vaccines in terms of serorotection/seroconversion rates. Also, as per the WHO requirement for vaccine development and pre-qualification, the lot-to-lot (LTL) consistency of three lots of hexavalent vaccine was evaluated^9^.

## Results

### Participants studied

**Fig. 1:**
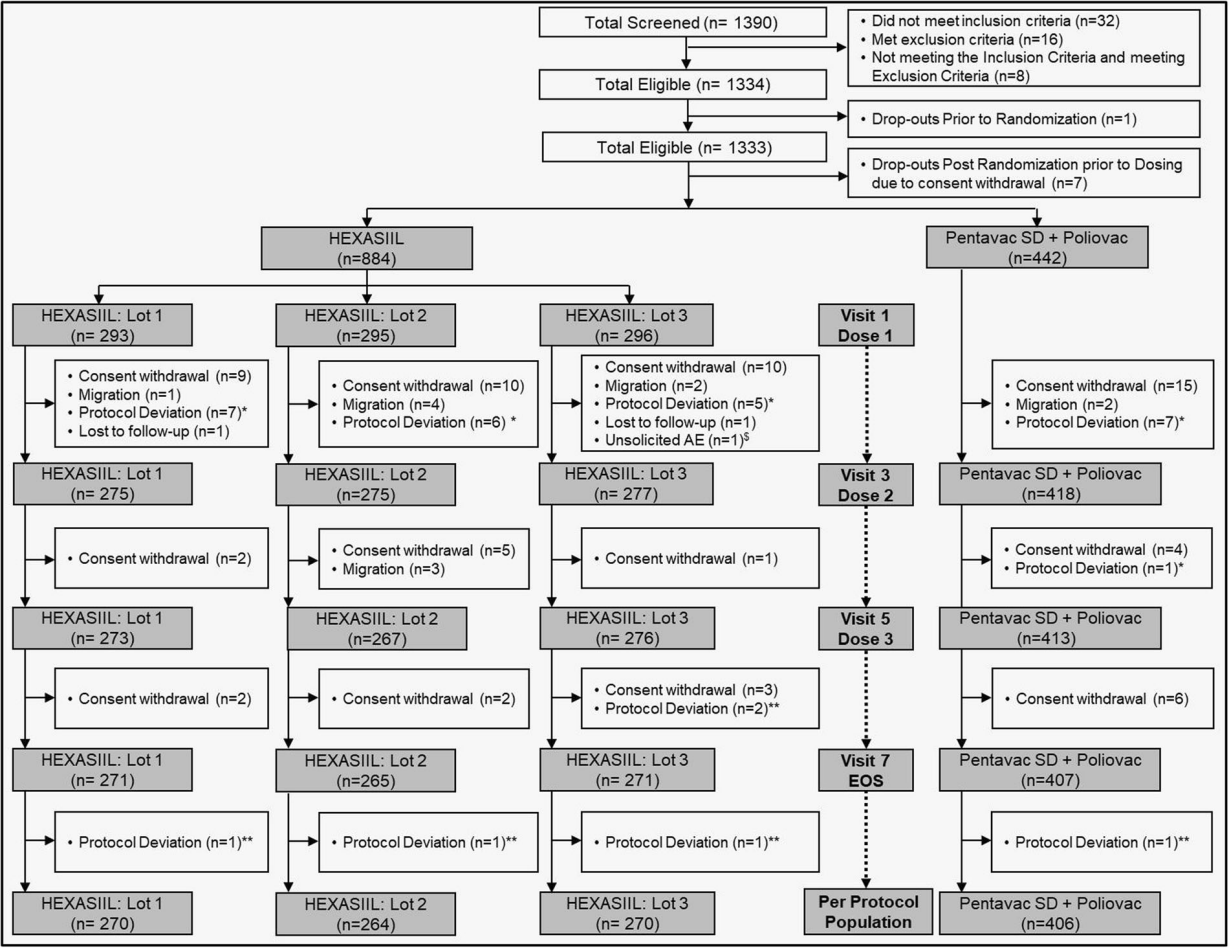
Subject disposition. n - number of subjects, EOS - End of Study, *Subjects received vaccines out of the study, ^$^One subject with SAE of Congenital adrenal hyperplasia discontinued, **Visit window deviation.

Overall, 1390 subjects were screened, of which 1334 met the eligibility criteria. Among those screened, 56 (4%) were screen failures. One subject was eligible, however, was dropped out prior to randomization as baseline immunogenicity sample was inadequate. Thus, a total of 1333 subjects were randomized such that 888 subjects were in the DTwP-HepB-IPV-Hib group and 445 subjects in DTwP-HepB-Hib + IPV group (Fig. 1). Among the 1333 randomized subjects, 7 dropped out prior to first vaccination due to consent withdrawal, thus a total of 1326 subjects received the first dose of the study vaccine such that 884 subjects (293 in Lot 1, 295 in Lot 2 and 296 in Lot 3) received DTwP-HepB-IPV-Hib vaccine and 442 received the comparator DTwP-HepB-Hib + IPV vaccines. Out of 1326, 289 subjects were enrolled before protocol amendment and 1037 were enrolled after protocol amendment. In DTwP-HepB-IPV-Hib group, 867 (98.1%) subjects and in DTwP-HepB-Hib + IPV group 430 (97.3%) subjects received at least one oral Rotavirus vaccine (ROTASIIL, Serum Institute of India Pvt. Ltd., India). A total of 804 (91.0%) subjects in the DTwP-HepB-IPV-Hib group and 406 (91.9%) subjects in the DTwP-HepB-Hib + IPV group were included in PP (per protocol) immunogenicity analysis. Demographic characteristics were similar in each group with a comparable proportion of male and female participants (Table 1). The PP population included subjects who received all study vaccines as per the assigned vaccine group and had pre- and post vaccination immunogenicity measurements with no major protocol deviations. This population served as the primary analysis population for the immunogenicity objectives.

**Table 1:** Baseline demography (safety population)

Demographics Characteristic	Statistic	DTwP-HepB-IPV-Hib (*N* = 884)	DTwP-HepB-Hib + IPV (*N* = 442)
Gender			
Male	*n* (%)	418 (47.3)	218 (49.3)
Female	*n* (%)	466 (52.7)	224 (50.7)
Age in Days	Mean (SD)	47.7 (4.13)	48.1 (4.06)
	Median (Min, Max)	47.0 (42, 56)	47.0 (42, 56)
Weight (kg)	Mean (SD)	4.27 (0.549)	4.31 (0.584)
	Median (Min, Max)	4.30 (2.4, 6.3)	4.30 (2.4, 5.9)
Height (cm)	Mean (SD)	54.32 (2.63)	54.50 (2.55)
	Median (Min, Max)	54.30 (45, 62)	55.00 (43, 61)

Percentage is based on Number of Subjects in the Safety Population for each study group

*SD* standard deviation

### Immunogenicity

Antibody thresholds and criteria used to define seroprotection/seroconversion rates were as per WHO Technical Report Series (TRS) for DT-based combined vaccines^10^. DTwP-HepB-IPV-Hib vaccine was found to be immunogenic with a robust immune response to all antigens. The lower limit of 95% confidence interval (CI) for the treatment difference for all antibodies was more than the defined lower limit of −10% (Table 2). Thus, DTwP-HepB-IPV-Hib vaccine demonstrated non-inferiority to the DTwP-HepB-Hib + IPV vaccines, in terms of seroprotection/seroconversion for all antigens.

**Table 2:** Non-inferiority assessment of Seroprotection/Seroconversion rates between DTwP-HepB-IPV-Hib and DTwP-HepB-Hib + IPV at 28 days post 3-dose of primary vaccination series in infants aged 6–8 weeks (Per Protocol population)

	Thresholds (Criteria for evaluation)	DTwP-HepB-IPV-Hib (*N* = 804)				DTwP-HepB-Hib + IPV (*N* = 406)				DTwP-HepB-IPV-Hib versus DTwP-HepB-Hib + IPV		NI met^b^
		*N*	*n*	%	95% CI	*N*	*n*	%	95% CI	Difference	95% CI	
Anti-D	≥0.1 IU/mL	804	801	99.6	98.9–99.9	406	405	99.8	98.6–100	−0.1	−2.2,2.0	Yes
Anti-T	≥0.1 IU/mL	804	804	100	99.5–100	406	406	100.0	99.1–100	0.0	NA	Yes
Anti-wP	>24 U/mL	804	603	75.0	71.9–78.0	406	309	76.1	71.7–80.2	−1.1	−6.0,3.8	Yes
Anti-PT	Seroconvesion^a^	804	648	80.6	77.7–83.3	406	324	79.8	75.6–83.6	0.8	−3.7,5.3	Yes
Anti-HBs	≥10 mIU/mL	804	787	97.9	96.6–98.8	406	393	96.8	94.6–98.3	1.1	−1.3,3.5	Yes
Anti-PRP	≥0.15 µg/mL	804	799	99.4	98.6–99.8	406	405	99.8	98.6–100	−0.4	−2.5,1.7	Yes
Anti-Polio 1	≥8 (1/dil)	796	795	99.9	99.3–100	402	398	99.0	97.5–99.7	0.9	−1.3,3.1	Yes
Anti-Polio 2	≥8 (1/dil)	796	791	99.4	98.5–99.8	402	399	99.3	97.8–99.8	0.1	−2.0,2.3	Yes
Anti-Polio 3	≥8 (1/dil)	796	795	99.9	99.3–100	402	399	99.3	97.8–99.8	0.6	−1.5, 2.8	Yes

*CI* Confidence Interval, *IU* International Unit, *NA* Not applicable, *NI* Non Inferiority

*N* Number of Subjects with a determinate antibody concentration/titre to the given antibodies

*n* Number of seroprotection/seroconversion subjects with Antigen/Serotype specific IgG Antibody Concentration/Titre ≥ Threshold (Criteria for Evaluation)

(%) [Number of seroprotection/seroconversion/Number of Subjects with a determinate antibody concentration/titre to the given Antigen/Serotype] *100

^a^Seronversion: In subjects with no quantifiable antibody - below lower limit of quantification (LLOQ) -prior to vaccination, seroconversion is defined as achieving a quantifiable antibody level post-vaccination. In subjects with quantifiable antibody prior to vaccination, seroconversion is defined by a 4-fold-increase in antibody titres from pre- to post-vaccination.

^b^For all antigens, non-inferiority concluded as the lower limit of 2-sided 95% CI of difference between groups is greater than −10%.

Geometric mean concentrations/titers (GMC/GMTs) for tetanus, pertussis toxin (PT), hepatitis B, polio type 1 and 3 antigens were higher in DTwP-HepB-IPV-Hib group compared to DTwP-HepB-Hib + IPV. While GMCs/GMTs for diphtheria, *B. Pertussis*, polio type 2 and anti-PRP were lower in the DTwP-HepB-IPV-Hib group compared to DTwP-HepB-Hib + IPV. Post 3rd dose, the GMC/GMT were significantly high for all antigens (*p* < 0.0001 as calculated using Mix Model) as compared to pre vaccination levels. Also, a higher geometric mean fold rise (GMFR) over the baseline was observed. The GMFR was statistically not significant between the groups for anti-D, anti-T, anti-*B. Pertussis*, anti-PT and anti-HBsAg antibodies. However, there was statistically significant difference in the GMFR for anti-PRP and anti-polio 1, 2, 3 antibodies (Table 3).

**Table 3:** GMC/GMT, GMFR at baseline and at 28 days post 3^rd^ dose of primary vaccination series in infants aged 6–8 weeks - PP Population

Antibody		DTwP-HepB-IPV-Hib (*N* = 804^a^)		DTwP-HepB-Hib + IPV (*N* = 406^a^)		
		Baseline	Post 3^rd^ dose^#^	Baseline	Post 3^rd^ dose^#^	GMFR p value^$^
Anti-D (IU/ml)	GMC	0.144 (0.131, 0.157)	0.968 (0.904, 1.036)	0.168 (0.148, 0.190)	0.969 (0.883, 1.063)	NA
	GMFR	NA	6.72 (6.088, 7.421)	NA	5.77 (5.038, 6.605)	0.6195
Anti-T (IU/ml)	GMC	1.018 (0.958, 1.083)	3.903 (3.712, 4.103)	1.192 (1.102, 1.289)	3.877 (3.622, 4.149)	NA
	GMFR	NA	3.83 (3.604, 4.072)	NA	3.25 (3.022, 3.499)	0.1008
Anti-wP (U/ml)	GMC	8.485 (7.923, 9.088)	46.445 (43.511, 49.576)	10.145 (9.176, 11.217)	49.839 (45.651, 54.413)	NA
	GMFR	NA	5.47 (5.044, 5.940)	NA	4.91 (4.386, 5.502)	0.6208
Anti-PT (IU/ml)	GMC	3.500 (3.350, 3.656)	38.840 (36.013, 41.890)	3.2697 (3.089, 3.460)	35.843 (31.774, 40.434)	NA
	GMFR	NA	11.10 (10.168, 12.111)	NA	10.96 (9.589, 12.533)	0.2441
Anti-HBs (mIU/ml)	GMC	2.087 (1.892, 2.302)	1783.552 (1577.500, 2016.518)	2.094 (1.837, 2.388)	1721.769 (1430.532, 2072.298)	NA
	GMFR	NA	854.61 (735.484, 993.041)	NA	822.07 (655.646, 1030.750)	0.7476
Anti-PRP (µg/ml)	GMC	0.310 (0.286, 0.336)	2.822 (2.551, 3.122)	0.371 (0.331, 0.417)	4.818 (4.216, 5.506)	NA
	GMFR	NA	9.09 (8.182, 10.103)	NA	12.96 (11.108, 15.123)	<0.0001
Anti-Polio 1 (1/dil)	GMT	210.055 (182.346, 241.975)	3215.314 (3008.131, 3436.768)	189.217 (155.240, 230.631)	2360.426 (2100.827, 2652.102)	NA
	GMFR	NA	15.08 (13.045, 17.438)	NA	12.31 (9.984, 15.188)	<0.0001
Anti-Polio 2 (1/dil)	GMT	14.074 (13.052, 15.177)	122.157 (111.840, 133.427)	13.430 (12.117, 14.884)	152.352 (136.314, 170.277)	NA
	GMFR	NA	8.68 (7.615, 9.899)	NA	11.45 (9.648, 13.589)	0.0023
Anti-Polio 3 (1/dil)	GMT	38.511 (33.270–44.577)	1955.913 (1824.171, 2097.168)	28.455 (23.391, 34.614)	1361.409 (1221.327, 1517.557)	NA
	GMFR	NA	50.79 (43.494, 59.299)	NA	48.23 (39.143, 59.435)	<0.0001

*CI* Confidence Interval, *dil* dilution, *N* per protocol population, *n* Number of subjects contributing to the analysis

^$^*p* values were calculated using Mix Model. ^#^Pre and post 3^rd^ dose GMT/GMC comparison for all antigens using paired *t* test has *p* < 0.0001. *p* < 0.05 was considered significant

^a^For Anti-Polio 1, 2 and 3 *N* = 796 each for DTwP-HepB-IPV-Hib group and *N* = 402 each for DTwP-HepB-Hib + IPV group.

LTL consistency between three lots of DTwP-HepB-IPV-Hib vaccine was demonstrated as the limits of 95% CI for the ratio of GMC/GMTs 28 days post 3rd dose for any pair of DTwP-HepB-IPV-Hib vaccine lots were within the pre-specified equivalence limits of 0.5 and 2 (Table 4).

**Table 4:** Equivalence of geometric mean concentrations/titers (GMC/GMTs) between three lots of DTwP-HepB-IPV-Hib at 28 days post 3^rd^ dose of primary vaccination series in infants aged 6–8 weeks - PP Population

Antibody	Lot 1 (*N* = 270^a^)		Lot 2 (*N* = 264^a^)		Lot 3 (*N* = 270^a^)		GMC/T ratio (95% CI)^b^		
	GMC/T	(95% CI)	GMC/T	(95% CI)	GMC/T	(95% CI)	Lot 1/ Lot 2	Lot 1/Lot 3	Lot 2/Lot 3
Anti-D (IU/ml)	0.867	(0.770, 0.977)	1.023	(0.908, 1.153)	1.022	(0.909, 1.149)	0.847 (0.716, 1.002)	0.848 (0.718, 1.002)	1.001 (0.847, 1.183)
Anti-T (IU/ml)	3.997	(3.657, 4.369)	3.782	(3.478, 4.113)	3.929	(3.597, 4.291)	1.056 (0.935, 1.194)	1.017 (0.897, 1.152)	0.962 (0.852, 1.087)
Anti-wP (U/ml)	44.212	(39.306, 49.730)	45.480	(40.462, 51.120)	49.802	(44.829, 55.327)	0.972 (0.823, 1.147)	0.887 (0.758, 1.039)	0.913 (0.780, 1.068)
Anti-PT (IU/ml)	37.321	(32.736, 42.548)	39.785	(34.793, 45.493)	39.483	(34.697, 44.930)	0.938 (0.778, 1.131)	0.945 (0.786, 1.135)	1.007 (0.836, 1.213)
Anti-HBs (mIU/ml)	1703.715	(1394.042, 2082.180)	1938.477	(1525.147, 2463.823)	1721.089	(1411.152, 2099.099)	0.879 (0.643, 1.201)	0.990 (0.747, 1.312)	1.126 (0.826, 1.537)
Anti-PRP (µg/ml)	2.825	(2.363, 3.378)	2.801	(2.349, 3.340)	2.841	(2.392, 3.373)	1.008 (0.785, 1.295)	0.994 (0.776, 1.273)	0.985 (0.771, 1.260)
Anti-Polio 1 (1/dil)	3180.539	(2835.895, 3567.068)	3233.812	(2889.801, 3618.774)	3232.618	(2866.445, 3645.567)	0.983 (0.837, 1.154)	0.983 (0.833, 1.161)	1.000 (0.848, 1.179)
Anti-Polio 2 (1/dil)	121.728	(104.942, 141.199)	148.507	(128.207, 172.019)	101.066	(86.021, 118.744)	0.819 (0.665, 1.009)	1.204 (0.968, 1.498)	1.469 (1.181, 1.826)
Anti-Polio 3 (1/dil)	2056.808	(1836.936, 2302.997)	1933.902	(1711.787, 2184.837)	1879.468	(1653.096, 2136.838)	1.063 (0.901, 1.255)	1.094 (0.922, 1.297)	1.028 (0.862, 1.227)

*CI* Confidence Interval, *IU* International Unit, *PRP* polyribosylribitol phosphate, *PT* Pertussis Toxin

*n* Number of subjects contributing to the analysis, *N* per protocol population

^a^For Anti-Polio 1, 2 and 3, for Lot 1 *N* = 269; for Lot 2 *N* = 262; for Lot 3 *N* = 265

^b^Equivalence concluded as the limits of 2-sided 95% CI of difference between lots are between the interval (0.5, 2.0).

### Safety

There were no unsolicited immediate adverse events (IAEs). There was one IAE each of pain and erythema in DTwP-HepB-IPV-Hib group and DTwP-HepB-Hib + IPV group. Additionally, one IAE of swelling at injection site was reported in DTwP-HepB-Hib + IPV group. The overall incidence of local solicited adverse events (AEs) was slightly higher in the DTwP-HepB-Hib + IPV group, but the differences were not statistically significant. The most frequently reported local AE in both the treatment groups was injection site pain followed by swelling and erythema. Most local AEs were of Grade 1 or Grade 2 severity. Grade 3 AEs were reported in 9.7% subjects in DTwP-HepB-IPV-Hib group and in 11.3% subjects in DTwP-HepB-Hib + IPV group, the difference being statistically not significant (Table 5). Overall, 654 (74%) subjects in the DTwP-HepB-IPV-Hib group and 333 (75.3%) subjects in DTwP-HepB-Hib + IPV group experienced one or the other systemic solicited AEs following vaccination. Irritability was the most frequently reported systemic solicited AE in both groups followed by crying, pyrexia, decreased appetite and somnolence. There were no significant differences between the two groups in the incidence of systemic solicited AEs. Most of the events were Grade 1 or Grade 2 in severity and were comparable between the two groups. Grade 3 events were comparable across two groups [DTwP-HepB-IPV-Hib group (2.7%) vs DTwP-HepB-Hib + IPV group (2.0%)] (Table 5). All local and systemic solicited AEs resolved completely without any sequelae and none led to any discontinuations from the study.

**Table 5:** Distribution of solicited adverse events during the study (Safety Analysis Set)

Participants with at least one:	DTwP-HepB-IPV-Hib (*N* = 884)		DTwP-HepB-Hib + IPV (*N* = 442)			% Difference (95% CI)	*p* value
	*n* (%)	95% CI		*n* (%)	95% CI		
Solicited Local AE	746 (84.4)	(81.8, 86.7)		386 (87.3)	(83.9, 90.3)	−2.9 (−7.0, 1.1)	0.161
Grade 1	693 (78.4)	(75.5, 81.1)		363 (82.1)	(78.2, 85.6)	−3.7 (−8.3, 0.9)	0.128
Grade 2	306 (34.6)	(31.5, 37.9)		151 (34.2)	(29.7, 38.8)	0.5 (−5.0, 5.9)	0.902
Grade 3	86 (9.7)	(7.9, 11.9)		50 (11.3)	(8.5, 14.6)	−1.6 (−5.0, 1.9)	0.388
Injection site erythema	265 (30.0)	(27.0, 33.1)		142 (32.1)	(27.8, 36.7)	−2.1 (−7.4, 3.1)	0.448
Injection site pain	694 (78.5)	(75.6, 81.2)		360 (81.4)	(77.5, 85.0)	−2.9, (−7.6, 1.7)	0.220
Injection site swelling	357 (40.4)	(37.1, 43.7)		180 (40.7)	(36.1, 45.5)	−0.3 (−5.9, 5.3)	0.905
Solicited Systemic AE	654 (74.0)	(71.0, 76.8)		333 (75.3)	(71.0, 79.3)	−1.4 (−6.3, 3.6)	0.640
Grade 1	630 (71.3)	(68.2, 74.2)		325 (73.5)	(69.2, 77.6)	−2.3 (−7.4, 2.9)	0.399
Grade 2	223 (25.2)	(22.4, 28.2)		91 (20.6)	(16.9, 24.7)	4.6 (−0.2, 9.5)	0.064
Grade 3	24 (2.7)	(1.7, 4.0)		9 (2.0)	(0.9, 3.8)	0.7 (−1.1, 2.5)	0.575
Vomiting	133 (15.0)	(12.8, 17.6)		63 (14.3)	(11.1, 17.9)	0.8 (−3.3, 4.8)	0.743
Crying	384 (43.4)	(40.1, 46.8)		186 (42.1)	(37.4, 46.8)	1.4 (−4.3, 7.0)	0.680
Irritability	465 (52.6)	(49.2, 55.9)		219 (49.5)	(44.8, 54.3)	3.1 (−2.7, 8.8)	0.294
Pyrexia	357 (40.4)	(37.1, 43.7)		165 (37.3)	(32.8, 42.0)	3.1 (−2.5, 8.6)	0.310
Decreased appetite	216 (24.4)	(21.6, 27.4)		91 (20.6)	(16.9, 24.7)	3.8 (−1.0, 8.7)	0.128
Somnolence	164 (18.6)	(16.0, 21.3)		85 (19.2)	(15.7, 23.2)	−0.7 (−5.1, 3.8)	0.765

*AE* Adverse Event, *CI* Confidence Interval

*n* Count of Subjects (at least one event i.e., Subjects counted only once if the Subject reported one or more Events), % (n/ Number of Subjects in Safety Population who received respected Dose)*100;

*p* values were calculated using Newcombe method.

The number of subjects reporting at least one unsolicited AE was comparable between the two groups viz., 294 (33.3%) subjects in the DTwP-HepB-IPV-Hib group and in 155 (35.1%) subjects in DTwP-HepB-Hib + IPV group. The maximum severity of most of the events was Grade 2; Grade 3 AEs were reported in 3 (0.3%) subjects in DTwP-HepB-IPV-Hib and 4 (0.9%) subjects in DTwP-HepB-Hib + IPV group. Upper respiratory tract infection was the most commonly reported unsolicited AE across both groups. Other common events were diarrhoea, rhinitis, nasopharyngitis, abdominal pain and fever reported with comparable frequency, among both the groups (Supplementary Table 3).

A total of 10 serious adverse events (SAEs) were reported up to 28 days post dose 3, of which 6 SAEs were reported in DTwP-HepB-IPV-Hib group and 4 in DTwP-HepB-Hib + IPV group; all SAEs met the seriousness criterion of hospitalization (Supplementary Table 4). All SAEs recovered without any sequelae except the event of Congenital Adrenal Hyperplasia reported in DTwP-HepB-IPV-Hib group, which was considered stabilized and the subject was discontinued. None of the SAEs was assessed as related to the study vaccine. No other AEs led to discontinuation or death.

## Discussion

This Phase III pivotal, licensure study was intended to evaluate the immunogenicity and safety of a new hexavalent vaccine i.e., DTwP-HepB-IPV-Hib group in comparison to DTwP-HepB-Hib + IPV group, in healthy infants. All these vaccines are manufactured by SIIPL. Pentavac SD and Poliovac vaccines are prequalified by WHO for purchase by United Nations agencies. The study endpoints were based on the WHO-TRS recommendations for DT based combined vaccines and WHO guidelines on clinical evaluation of vaccines^10,11^. Non-inferiority of DTwP-HepB-IPV-Hib vaccine compared to licensed vaccines DTwP-HepB-Hib + IPV was demonstrated with respect to seroprotection/seroconversion for all vaccine antigens. Overall, the immunogenicity data showed a robust response to each antigen following a 3-dose of primary vaccination series of DTwP-HepB-IPV-Hib vaccine, in infants. Achievement of a robust immune response to D, T, P, HepB, Hib and Polio after the primary vaccination is critical to protect children in their first year of life, before a booster dose is administered. Also, the reliability of manufacturing process is confirmed with the demonstration of a lot-to-lot consistency of three batches of DTwP-HepB-IPV-Hib vaccine. The well established immunological correlates were used for assessment of responses to D, T, Hep B, IPV and Hib. For pertussis no serological correlate of protection has been established^12^. PT is the exclusive antigen for *B. Pertussis* and is important for pathogenicity^13,14^. The antibodies against PT mainly contribute to protection against pertussis and WHO recommends the assessment of PT response by ELISA with wP based vaccine also^10^. Therefore, anti-PT response was selected for the evaluation of the pertussis response using a CE certified PT specific ELISA kit, and were included in the main statistical analyses along with anti *B. Pertussis* IgG assessed using mixed antigen commercial ELISA kit. The seroprotection/seroconversion rates with DTwP-HepB-IPV-Hib vaccine were comparable to that reported for other whole cell hexavalent combination vaccines^15,16^ and in previous studies with SIIPL’s DTwP-HepB-Hib vaccine^15,17,18^. Although statistically significant difference in GMFR was observed between the groups for PRP and IPV antigens, it is not considered clinically relevant, as antibody titers for these antigens are well above the seroprotective level in almost all subjects and primary endpoint of non-inferiority for seroprotection/seroconversion rates was clearly met. Moreover, these findings are in line with those for other wP based hexavalent vaccines^15,16^.

No safety concerns were observed with administration of the DTwP-HepB-IPV-Hib vaccine in this study, and safety profile was in-line with that reported for other wP-containing hexavalent^15,16^ and pentavalent vaccines^17,18^. No clinically significant difference in the incidence of solicited local and systemic AEs was reported between the two vaccine groups. None of the SAEs was causally related to the vaccine, in both the groups. There were no safety concerns associated with co-administration of oral Rotavirus vaccine, consistent with studies done for concomitant administration of DT based hexavalent^16^ and pentavalent^19^ vaccines with oral rotavirus vaccines.

Cases of vaccine-associated paralytic poliomyelitis and circulating vaccine-derived poliovirus type 2 (cVDPV2) are occasionally reported in countries where wild poliovirus has been eliminated, and therefore moving from OPV to IPV is very important in complete global eradication of polio, and vaccines combining IPV appear to be one of the potential approaches to facilitate this change^20^. The importance of introduction of IPV is reflected in WHO guidelines recommending the administration of at least one IPV dose to all children^21^. The availability of an affordable ultimate combination vaccine providing robust immune response against type 1, 2, and 3 polioviruses will help to deliver promise of polio-free world set as per the recent Global Polio Eradication strategy^22,23^. The use of wP in hexavalent vaccines is important, especially in developing-country settings, both because of cost and rising concerns of resurgence due to shorter duration of effectiveness of acellular pertussis (aP) vaccines. Also, WHO has recommended that national programmes currently administering wP vaccination should continue to use wP vaccines for primary vaccination series^24^. The hexavalent vaccine represents an alternative to current schedules of pentavalent and standalone IPV vaccines and the need for fewer vaccination sessions, reduce logistics costs associated with immunization programs and potentially higher coverage. Another significant advantage of this vaccine is less risk premature discontinuation of IPV from immunization programs^25^. A wP based hexavalent combination vaccine could be administered using the schedules currently recommended for the pentavalent vaccine (i.e., at 8, 12 and 16 weeks, or 6, 10 and 14 weeks, plus a booster dose at least 6 months later)^21^.

The limitation of the study is the difference in the vaccine administration between the two study groups resulting in a challenge in employing a double-blind or an observer-blind study design. Therefore, the study was designed as an open-label study which could have introduced bias in the reporting of safety. The immune response to Rotavirus vaccine antigens were not measured is other limitation of the study.

In conclusion, the immunogenicity data showed a robust response to each antigen following a 3-dose primary series of DTwP-HepB-IPV-Hib vaccine in infants. Non-inferiority and lot-to-lot consistency for DTwP-HepB-IPV-Hib vaccine was demonstrated. There were no alarming safety observations and vaccine was generally well-tolerated. Assured consistent supply of this hexavalent vaccine could be considered a feasible choice in the shift from OPV to IPV vaccination, particularly in developing countries.

## Methods

### Study design and participants

This was a combined Phase II/III open label, randomized, active-controlled study in healthy toddlers and infants carried out at 18 study sites in India between February, 2020 and March, 2021. In Phase II part of the study, healthy toddlers were randomized to receive a single booster dose of study vaccines DTwP-HepB-IPV-Hib vaccine or DTwP-HepB-Hib + IPV, at 12–24 months of age. The safety data from the Phase II study was reviewed by an independent DSMB (Data Safety Monitoring Board) and following positive recommendation from the DSMB, the Phase III study in infants was initiated. We plan to report outcomes of the Phase II part in a separate publication. The study protocol and its amendment was reviewed and approved by the Institutional Review Board (IRB)/Institutional Ethics Committee (IEC) responsible for the respective study sites (Supplementary Table 1) and the Drug Controller General of India (DCGI). The trial was registered on the national clinical trial registry [Clinical Trials Registry India (CTRI) Number - CTRI/2019/11/022052]. The study was performed in accordance with New Drugs and Clinical Trials Rules (2019), National Ethical Guidelines for Biomedical and Health Research Involving Human Participants, Indian Council of Medical Research Guidelines, International Council on Harmonisation Guideline for Good Clinical Practice (ICH-GCP) and the Declaration of Helsinki.

Healthy infants aged 6–8 weeks (42 to 56 days, both days inclusive), born at full term pregnancy (≥37 weeks) who had received the birth doses of oral poliovirus vaccine (OPV) and Bacillus Calmette-Guérin (BCG) at least 4 weeks before the first trial vaccination were eligible. Written informed consent was obtained from parent/s of eligible subjects. The main exclusion criteria were history of diphtheria/tetanus/pertussis/hepatitis B/*H. Influenzae type b*/poliomyelitis infection(s) (confirmed either clinically, serologically or microbiologically); previous vaccination or planned receipt of any vaccine against D, T, P, Hep B (except birth dose), poliomyelitis (except OPV) or *H. Influenzae type b*, apart from trial vaccines during the study period; administration of any vaccine (except OPV during government immunization campaign) in the 4 weeks preceding the first trial vaccination; history of major congenital defects or illness; history of anaphylaxis to any vaccine or components of study vaccine; known or suspected impairment of the immune function, or those who received immunosuppressive therapy; presence of evolving or changing neurological disorder or history of seizures and/or encephalopathy; known personal or maternal history of HIV, Hepatitis B or Hepatitis C seropositivity; receipt of blood or blood-derived products or immunoglobulins or planned administration during the trial; history of any clinically significant chronic disease that in the opinion of the Investigator, might interfere with the evaluation of the study objectives.

Eligible infants were randomized in a 2:1 ratio to receive either DTwP-HepB-IPV-Hib vaccine or the comparator viz., DTwP-HepB-Hib + IPV vaccines. Further, the subjects in DTwP-HepB-IPV-Hib vaccine group were randomized in a 1:1:1 ratio to receive any one of the 3 lots (Lot 1, 2 or 3) of DTwP-HepB-IPV-Hib vaccine. The computer-generated randomization list for vaccine assignment was generated by the contract research organization before the start of the study and randomization was done through interactive web response systems. All infants randomized to the DTwP-HepB-IPV-Hib vaccine group received 0.5 mL dose as an intramuscular injection in the upper anterolateral aspect of the thigh, while infants randomized to the comparator group received 0.5 mL DTwP-HepB-Hib vaccine and 0.5 mL of IPV vaccine, administered as two intramuscular injections at upper antero-lateral aspects of right and left thigh respectively. Vaccination schedule consisted three doses using 6, 10, and 14 weeks schedule.

### Study vaccines

DTwP-HepB-IPV-Hib (HEXASIIL), vaccine (Lot numbers, 2649 × 003 [Lot 1, Single dose vial], 2659 × 004 [Lot 2, Multiple dose (10-dose) vial] and 2649K002 [Lot 3, Single dose Pre-filled syringe]) manufactured by SIIPL was used. Each 0.5 ml dose contained Diphtheria Toxoid ≥30 IU, Tetanus Toxoid ≥40 IU, *B. pertussis* (whole cell) ≥4 IU, HBsAg (rDNA) 15 mcg, Hib conjugate (PRP-TT) 10 mcg, 40, 8 and 32 D antigen units of poliovirus (Salk strains grown on vero cells) type 1 (Mahoney strain), type 2 (MEF-1 strain) and type 3 (Saukett strain) respectively, and Aluminium Phosphate gel ≤1.25 mg, as an adjuvant.

DTwP-HepB-Hib vaccine (Pentavac^**®**^SD, Batch no. 2859L008D) manufactured by SIIPL was available as a homogenous liquid in a single dose vial. Each 0.5 ml dose contained Diphtheria Toxoid ≥30 IU, Tetanus Toxoid ≥40 IU, *B. pertussis* (whole cell) ≥4 IU, HBsAg (rDNA) ≥ 10 mcg, Hib conjugate (PRP-TT) 10 mcg and Aluminium Phosphate ≤1.25 mg, as an adjuvant.

IPV (Poliovac^**®**^, batch no. 1519T004B) manufactured by SIIPL was a sterile suspension in a single dose vial. Each 0.5 mL dose contained 40, 8 and 32 D antigen units of Vero cell cultivated poliovirus type 1 (Mahoney strain), type 2 (MEF-1 strain), and type 3 (Saukett strain), respectively.

### Serology

Blood sample (approximately 5 ml) was collected prior to first vaccination and 28 days post third dose of primary vaccination series, for immunogenicity assessments. Anti-diphtheria, anti-tetanus, anti-Pertussis Toxin (PT), anti- *B. Pertussis*, anti-HBsAg, and anti-PRP antibody titres were measured by Enzyme Linked Immunosorbent Assays (ELISA) at Metropolis Healthcare Ltd., Mumbai, a Good Laboratory Practice (GLP) certified, CAP (College of American Pathologists) and NABL (National Accreditation Board for Testing & Calibration Laboratories) accredited laboratory in India. Antibodies testing against diphtheria, tetanus and pertussis was performed using commercial CE certified kits (RE56191 for diphtheria, RE56901 for tetanus and RE56141 for pertussis; IBL International GmbH, Germany). The kits were validated using international reference standards as per ICH, US FDA and EMEA guidance on bioanalytical methods. The kit method is based on direct ELISA method wherein the IgG antibodies in sera sample is captured using a purified antigen and detected using an IgG specific detection antibody. Reference standard used in the kits are calibrated against the international standard and use four parameter logistic curve fit program to report the unitage in IU/mL. For, diphtheria and tetanus, seroprotection was defined as an anti-diphtheria and anti-tetanus antibodies titer level of ≥0.1 Iµ/ml. A whole-cell ELISA was used to detect antibodies to *B. pertussis*. The commercial kit used for pertussis was coated with *B. Pertussis antigens* (containing PT, Filamentous hemagglutinin and lipopolysaccharides) and standardized in U/ml. There is no international standard definition for seroprotection for *B. pertussis*. As per the standard curve provided in the kit literature the quantitative threshold of 24 µ/ml was used to interpret the results for anti *B. Pertussis* IgG. The anti-PT antibodies were measured by ELISA using CE certified commercial kit (Euroimun, Germany). Results for anti-PT antibody were analyzed using lower limit of quantitation (5 Iµ/ml). The Hib ELISA specifically detects antibodies against PRP, and the seroprotection was considered as anti PRP antibody concentration ≥0.15 µg/ml. Antibodies against hepatitis B were determined by ELISA and seroprotection was defined as concentration of anti-HepB antibodies ≥10 mIµ/ml. The VaccZyme^TM^ (MK016.U; Binding Site Group Ltd., United Kingdom) and ARCHITECT Anti-HBs (Abbott Laboratories, Ireland) commercial kits were used for antibodies against Hib and Hepatitis B, respectively. Anti-polio type 1, 2 & and 3 antibody titres were measured by Neutralization assay against sabin type poliovirus strains at Viroclinics Biosciences B.V. (Rotterdam, The Netherlands), which is GLP certified laboratory and accredited by, Raad voor Accreditatie (RvA), the Dutch Accreditation Council. Seroconversion was defined anti-polio 1, 2 and 3 titers ≥8 (1/dil).

### Safety and reactogenicity

After administration of each vaccine dose, all subjects were observed for a minimum of 30 min at the study clinic to record any IAEs. At the end of 30-min observation period, vital signs and targeted physical examination (if indicated) were assessed. During the first 7 days post each vaccination, subjects were assessed for any local (pain/tenderness, erythema, swelling) and systemic solicited AEs (fever [defined as a body temperature ≥38 °C/100.4 °F as measured by axillary route], irritability, abnormal crying, drowsiness, vomiting, loss of appetite). Unsolicited AEs and SAEs were collected throughout the study starting from signing the informed consent, till 28 days following vaccination. The parent(s) were given subject diary card to record details of solicited and unsolicited AEs and to capture medication details. Severity of all solicited AEs was graded as per the clinical judgment of the investigator considering information provided by parents, and the guidance provided (Supplementary Table 2) in the protocol. The severity of all unsolicited AEs and SAEs assessed by the investigator as Grade 1/Mild (transient or mild discomfort <48 h; no medical intervention required), Grade 2/Moderate (mild to moderate limitation in activity, no or minimal medical intervention required), Grade 3/Severe (marked limitation in activity, hospitalization possible), Grade 4 (Life Threatening) and relationship to vaccination was assigned.

### Statistical analyses

The primary objective was to demonstrate the non-inferiority of DTwP-HepB-IPV-Hib vaccine in comparison with DTwP-HepB-Hib + IPV in terms of seroprotection rates for diphtheria, tetanus, Hepatitis B, *H. Influenzae* type b and seroconversion for poliovirus types 1, 2 and 3 and Pertussis Toxoid, 28 days following the third dose of study vaccine. Equivalence of immunogenicity of 3 lots of DTwP-HepB-IPV-Hib vaccine was a secondary endpoint for the study. As per fixed-sequence method strategy adopted for this study, LTL comparison was performed only if primary objective of NI was demonstrated, hence adjusting for multiplicity was not required.

For each antigen/serotype, NI was shown if a two-sided 95% confidence interval (CI) for the absolute difference in response proportions in terms of seroprotection/seroconversion [proportion of responders with DTwP-HepB-IPV-Hib vaccine minus proportion with Comparator (DTwP-HepB-Hib + IPV)] has lower limit > −10%. The sample size was chosen in an iterative, trial-and-error fashion to give the desired power of at least 90%. First, the sample size for LTL consistency was derived and then adjusted with respect to NI for 2:1 allocation ratio (DTwP-HepB-IPV-Hib vaccine: Comparator vaccines). The sample size of 250 evaluable subjects in each lot of DTwP-HepB-IPV-Hib vaccine provided at least 96% power to demonstrate equivalence of all antigens across all 3 lots, if actual GMC ratio assumed as 1 and log_10_SD as 0.7. The sample size of 750 evaluable subjects in DTwP-HepB-IPV-Hib group and 375 evaluable subjects in Comparator group provided at least 95% power to test NI for all antigens assuming no difference in the actual seroprotection and seroconversion proportion and one-sided α of 2.5% and based on the previously reported seroprotection/seroconversion rates for DTwP-HepB-Hib vaccine^10^. This provided ~91% overall power for the total study. Power calculations were done using PASS software. Assuming dropout rate of 10%, the total sample size for testing the primary objective of NI was 1260 in the allocation ratio 2:1 i.e., 840 subjects of DTwP-HepB-IPV-Hib (280 in each of 3 lots) and 420 subjects of Comparator vaccine group.

Due to the COVID-19 pandemic and country wide lockdown, subjects who could not complete the primary vaccination series as per schedule or even within ≤6 months of age or subjects who received the primary vaccination outside the study were not considered for primary and secondary analysis using PP Population. To bridge this gap and maintain the study power to 90%, additional subjects were enrolled. The randomization schedule was generated with additional numbers (considering 10% dropout rate) than to be randomized (*N* = 1260), so it was not generated again and same was used to randomize additional subjects.

For NI testing, two-sided 95% CI for proportion of responders for each antigen/serotype for each study group was calculated using Clopper-Pearson method. Two-sided 95% CI for difference in proportion of responders for each antigen/serotype between two study groups was calculated by Farrington and Manning method.

GMCs/GMTs were summarized for all antigens/serotypes in each lot of DTwP-HepB-IPV-Hib, to check LTL consistency with corresponding two-sided 95% CIs based on t-distribution to provide population estimates. Two-sided 95% CIs for the ratios of GMCs/GMTs were constructed using log normal distribution. Pre and post 3rd dose GMT/GMC comparison for all antigens was done using *t* test. GMFR were compared and *p* values were provided using mix model with values < 0.05 considered statistically significant. LTL would be demonstrated if the lower and upper limits of the two-sided 95% CIs for GMC/GMT ratio between each pair among the 3 lots of DTwP-HepB-IPV-Hib was within the pre-defined equivalence limits of [0.5 to 2] for all antigens/serotypes.

Comparison of safety of DTwP-HepB-IPV-Hib vaccine with the comparator vaccines was the secondary objective. For solicited AEs, two-sided 95% exact CIs for each of the proportions was provided; also, two-sided 95% CIs for the difference between the proportions in subjects from both vaccine group was provided, along with *p* values using Newcombe method. Unsolicited AEs and SAEs were summarized by System Organ Class and Preferred Term using the Medical Dictionary for Regulatory Activities (MedDRA) dictionary version 22.1. Concomitant medications were coded using WHO Drug Dictionary (WHODD) version 2019. Demographic baseline characteristics were analyzed descriptively by vaccine group. Mean, standard deviation, median, and minimum and maximum values were calculated for quantitative variables, and frequencies and percentages were calculated for categorical variables. All statistical analyses were performed using SAS^®^ software version 9.4 (SAS Institute Inc, Cary, North Carolina, USA).

### Reporting summary

Further information on research design is available in the Nature Research Reporting Summary linked to this article.

### Supplementary information

Supplementary material

REPORTING SUMMARY

## Data Availability

All data upon which conclusions are drawn are included in the manuscript or in the supplemental information file provided.
