# Supplementary material for: A phase III randomized-controlled study of safety and immunogenicity of DTwP-HepB-IPV-Hib vaccine (HEXASIIL®) in infants
Source: NPJ Vaccines. 2024 Feb 22;9:41. doi: 10.1038/s41541-024-00828-w (PMC10881502; doi:10.1038/s41541-024-00828-w)
Supplement: Supplementary file 1 — Supplementary material [file 41541_2024_828_MOESM1_ESM.pdf]

Supplementary data for Hitt Sharma et al.

A phase III randomized-controlled study of safety and immunogenicity of DTwP-HepB-IPV-Hib vaccine (HEXASIIL®) in infants

Table of Contents

Table 1. List of study sites and Ethics Committees....................2
Table 2. Severity Grading of Local and Systemic Solicited Events....................3
Table 3. Unsolicited Adverse Events by Preferred Term and Severity (Safety population)....................4
Table 4. Summary of Serious Adverse Events....................6

Supplementary Table 1. : List of study sites and Ethics Committees

No	Site name	Ethics committee details
1	Acharya Vinoba Bhave Rural Hospital, Wardha	Institutional Ethics Committee of DMIMS Datta Meghe Institute of Medical Sciences Sawangi (Meghe) Wardha
2	Baramati Hospital, Baramati	KMRFs Nikop Institutional Ethics Committee
3	Bharati Vidyapeeth (Deemed to be University) Medical College & Hospital, Pune	Institutional Ethics Committee, Bharati Vidypeeth Deemed University
4	Government Medical College and Hospital, Nagpur	Institutional Ethics Committee,GMC Nagpur
5	Government Medical College and District General Hospital, Chandrapur	lnstitutional Ethics Committee GMC Chandrapur
6	Grant Medical Foundation Ruby Hall Clinic	Institutional Ethics Committee, Poona Medical Research Foundation, Pune
7	Hamdard Inst.of Medical Sci.and Research and Asso. Hakeem Abdul Hameed Centenary Hospital, New Delhi	Jamia Hamdard Institutional Ethics Committee
8	Institute of Child Health, Kolkata	Institutional Ethics Committee, Institute of Child Health
9	JSS Hospital, Mysore	Institutional Ethics Committee, JSS Medical College
10	KEM Hospital Research Centre, Pune	KEM Hospital Research Centre Ethics Committee
11	Mahatma Gandhi Mission's Medical College and Hospital, Aurangabad	Ethics Committee For Research On Human Subjects, MGM Aurangabad
12	Kasturba Medical College, Manipal	Manipal Academy of Higher Education Ethics Committee
13	Maulana Azad Medical College and Associated Lok Nayak Hospital, New Delhi	Institutional Ethics Committee, Maulana Azad Medical College & Associated Lok Nayak Govind Ballabh Pant Hospitals & Guru Nanak Eye Center
14	Niloufer Hospital, Hyderabad	Institutional Ethics Committee Osmania Medical College Osmania Medical College,Koti, Hyderabad
15	Pulse Multispeciality Hospital, Pune	Ethics Committee of Pulse Multispeciality Hospital, Pune
16	Sanjeevani Children's Hospital, Aurangabad	Aurangabad Health Care and Research LlP-Independent Ethics Committee
17	Sir Sayajirao General Hospital, Vadodara	Institutional Ethics Committee for Human Research, Baroda Medical College, Vadodara
18	Sri Ramachandra Hospital, Chennai	Institutional Ethics Committee, Sri Ramachandra Institute of Higher Education and Research (DU)

Supplementary Table 2. Severity Grading of Local and Systemic Solicited Events

	**Adverse Event Grade**		
	**Mild (Grade 1)**	**Moderate (Grade 2)**	**Severe (Grade 3)**
**Local Solicited Adverse Events**			
Injection site pain/ tenderness	Minor reaction when injection site is touched	Cries or protests when injection site is touched	Cries when injected limb is moved or spontaneously painful
Injection site Erythema*/ Redness*	> 0 to < 2.5 cm	≥ 2.5 to < 5.0 cm	≥ 5 cm
Injection site Swelling*	> 0 to < 2.5 cm	≥ 2.5 to < 5.0 cm	≥ 5 cm
**Systemic Solicited Adverse Events**			
Fever**	38.0°C to 38.5°C	≥ 38.5°C to < 39.5°C	≥ 39.5°C
Irritability	Easily consolable	Requiring increased attention	Inconsolable
Abnormal Crying***	< 1 hour	1 to 3 hours	> 3 hours
Drowsiness	Unusually sleepy	Not interested in surroundings or did not wake up for a meal	Sleepy most of the time or difficult to wake up
Vomiting	1 episode/day	2 to 5 episodes/day	≥ 6 episodes /day or requiring parenteral hydration
Loss of Appetite	Eating less than normal in quantity	Missed 1 to 2 meals/feeds	Missed ≥ 3 meals/feeds

*Measure and record the greatest diameter.

**For temperature measurement, the axillary route to be used.

*** Crying which is prolonged, persistent, unusual, increased as well as high-pitched, protracted uncontrollable, and inconsolable is to be considered as abnormal.

Supplementary Table 3. Unsolicited Adverse Events by Preferred Term and Severity (Safety population)

Adverse Event	DTwP-HepB-IPV-Hib (N=884) n (%)	DTwP-HepB-Hib + IPV (N=442) n (%)
Any Unsolicited AEs	294 (33.3)	155 (35.1)
Upper respiratory tract infection	148 (16.7)	59 (13.3)
Grade 1 (Mild)	32 (3.6)	9 (2.0)
Grade 2 (Moderate)	121 (13.7)	52 (11.8)
Diarrhoea	65 (7.4)	35 (7.9)
Grade 1 (Mild)	21 (2.4)	11 (2.5)
Grade 2 (Moderate)	44 (5.0)	25 (5.7)
Rhinitis	40 (4.5)	20 (4.5)
Grade 1 (Mild)	5 (0.6)	0
Grade 2 (Moderate)	36 (4.1)	20 (4.5)
Nasopharyngitis	25 (2.8)	11 (2.5)
Grade 1 (Mild)	18 (2.0)	11 (2.5)
Grade 2 (Moderate)	8 (0.9)	1 (0.2)
Abdominal pain	21 (2.4)	13 (2.9)
Grade 1 (Mild)	11 (1.2)	7 (1.6)
Grade 2 (Moderate)	11 (1.2)	6 (1.4)
Pyrexia	16 (1.8)	11 (2.5)
Grade 1 (Mild)	11 (1.2)	2 (0.5)
Grade 2 (Moderate)	6 (0.7)	9 (2.0)
Grade 3 (Severe)	0	1 (0.2)
Gastroenteritis	16 (1.8)	8 (1.8), 9
Grade 1 (Mild)	3 (0.3)	2 (0.5)
Grade 2 (Moderate)	14 (1.6)	7 (1.6)
Nasal congestion	15 (1.7)	9 (2.0)
Grade 1 (Mild)	15 (1.7)	9 (2.0)
Lower respiratory tract infection	18 (2.0)	3 (0.7)
Grade 1 (Mild)	1 (0.1)	0
Grade 2 (Moderate)	17 (1.9)	3 (0.7)
Constipation	7 (0.8)	7 (1.6)
Grade 1 (Mild)	1 (0.1)	1 (0.2)
Grade 2 (Moderate)	6 (0.7)	6 (1.4)

Adverse Event	DTwP-HepB-IPV-Hib (N=884)<br>n (%)	DTwP-HepB-Hib + IPV (N=442)<br>n (%)
Dermatitis	5 (0.6)	8 (1.8)
Grade 1 (Mild)	0	4 (0.9)
Grade 2 (Moderate)	5 (0.6)	4 (0.9)
Rash	10 (1.1)	3 (0.7)
Grade 1 (Mild)	6 (0.7)	1 (0.2)
Grade 2 (Moderate)	4 (0.5)	2 (0.5)
Vomiting	5 (0.6)	5 (1.1)
Grade 1 (Mild)	2 (0.2)	1 (0.2)
Grade 2 (Moderate)	3 (0.3)	4 (0.9)

n (%), E: n = Count of Subjects (at least one event i.e. Subjects counted only once if the Subject reported one or more Events), % = (n / Number of Subjects in Safety Population who received respected Dose) *100, E = Count of Events (Subject may be counted more than once)

Supplementary Table 4. Summary of Serious Adverse Events (safety population)

AE	HEXASIIL (N=884)<br>n (%), E	Pentavac SD + Poliovac (N=442)<br>n (%), E
Any SAEs	6 (0.7), 6	4 (0.9), 4
Adrenogenital syndrome	1 (0.1), 1	0
Pyrexia	0	1 (0.2), 1
Abscess	0	1 (0.2), 1
Aspiration	1 (0.1), 1	0
Pulmonary arterial hypertension	0	1 (0.2), 1
Lymphadenitis	1 (0.1), 1	0
Lower respiratory tract infection	1 (0.1), 1	0
Pneumonia	1 (0.1), 1	0
Upper respiratory tract infection	1 (0.1), 1	0
Testicular torsion	0	1 (0.2), 1

n (%), E: n = Count of Subjects (at least one event i.e., Subjects counted only once if the Subject reported one or more Events), % = (n / Number of Subjects in Safety Population) *100, E = Count of Events (Subject may be counted more than once)
