# Supplementary material for: A phase III randomized-controlled study of safety and immunogenicity of DTwP-HepB-IPV-Hib vaccine (HEXASIIL®) in infants
Source: NPJ Vaccines. 2024 Feb 22;9:41. doi: 10.1038/s41541-024-00828-w (PMC10881502; doi:10.1038/s41541-024-00828-w)
Supplement: Supplementary file 2 — REPORTING SUMMARY [file 41541_2024_828_MOESM2_ESM.pdf]

nature portfolio

Corresponding author(s): Dr Hitt Sharma
Last updated by author(s): Feb 5, 2024

Reporting Summary

Nature Portfolio wishes to improve the reproducibility of the work that we publish. This form provides structure for consistency and transparency in reporting. For further information on Nature Portfolio policies, see our Editorial Policies and the Editorial Policy Checklist.

Statistics

For all statistical analyses, confirm that the following items are present in the figure legend, table legend, main text, or Methods section.

n/a	Confirmed	
☐	☒	The exact sample size (*n*) for each experimental group/condition, given as a discrete number and unit of measurement
☐	☒	A statement on whether measurements were taken from distinct samples or whether the same sample was measured repeatedly
☐	☒	The statistical test(s) used AND whether they are one- or two-sided<br>*Only common tests should be described solely by name; describe more complex techniques in the Methods section.*
☐	☒	A description of all covariates tested
☐	☒	A description of any assumptions or corrections, such as tests of normality and adjustment for multiple comparisons
☐	☒	A full description of the statistical parameters including central tendency (e.g. means) or other basic estimates (e.g. regression coefficient) AND variation (e.g. standard deviation) or associated estimates of uncertainty (e.g. confidence intervals)
☐	☒	For null hypothesis testing, the test statistic (e.g. *F*, *t*, *r*) with confidence intervals, effect sizes, degrees of freedom and *P* value noted<br>*Give P values as exact values whenever suitable.*
☒	☐	For Bayesian analysis, information on the choice of priors and Markov chain Monte Carlo settings
☒	☐	For hierarchical and complex designs, identification of the appropriate level for tests and full reporting of outcomes
☒	☐	Estimates of effect sizes (e.g. Cohen's *d*, Pearson's *r*), indicating how they were calculated

Our web collection on statistics for biologists contains articles on many of the points above.

Software and code

Policy information about availability of computer code

Data collection	The data was recorded in the EDC system InForm version 6.3.1.2.
Data analysis	All statistical analyses were performed using SAS® software version 9.4 (SAS Institute Inc, Cary, North Carolina, USA).

For manuscripts utilizing custom algorithms or software that are central to the research but not yet described in published literature, software must be made available to editors and reviewers. We strongly encourage code deposition in a community repository (e.g. GitHub). See the Nature Portfolio guidelines for submitting code & software for further information.

Data

Policy information about availability of data

All manuscripts must include a data availability statement. This statement should provide the following information, where applicable:

- Accession codes, unique identifiers, or web links for publicly available datasets
- A description of any restrictions on data availability
- For clinical datasets or third party data, please ensure that the statement adheres to our policy

All data upon which conclusions are drawn are included in the manuscript or in the supplemental information file provided.

Research involving human participants, their data, or biological material

Policy information about studies with human participants or human data. See also policy information about sex, gender (identity/presentation), and sexual orientation and race, ethnicity and racism.

Reporting on sex and gender	Sex is reported in the study. Findings apply to both males and females. Sex distribution is provided in the Table 1 of the manuscript. Sex based analyses were not performed as there are no differences in protection conferred by immunization in boys and girls.
Reporting on race, ethnicity, or other socially relevant groupings	Race, ethnicity, or other socially relevant groupings were not reported.
Population characteristics	Healthy infants aged 6-8 weeks (42 to 56 days, both days inclusive), born at full term pregnancy (≥ 37 weeks) who had received the birth doses of OPV and BCG at least 4 weeks before the first trial vaccination were included.
Recruitment	The computer-generated randomization list for vaccine assignment was generated by the contract research organization before the start of the study and randomization was done through interactive web response systems.
Ethics oversight	1. KEM Hospital Research Centre, Pune 2. Kasturba Medical College, Manipal 3. Sri Ramachandra Institute of Higher Education and Research, Chennai 4. JSS Medical College Hospital, Mysore 5. Bharati Vidyapeeth (Deemed to be University) Medical college & Hospital, Pune 6. Maulana Azad Medical College, New Delhi 7. Hamdard Institute of Medical Science & Research, New Delhi 8. Institute of Child Health, Kolkata 9. Acharya Vinoba Bhave Rural Hospital, Wardha 10. Government Medical College and Hospital, Nagpur 11. Baramati Hospital, Baramati, 12. Pulse Multispeciality Hospital, Pune 13. Mahatma Gandhi Mission hospital, Aurangabad 14. Sir Sayajirao General Hospital, Vadodara 15. Niloufer Hospital, Hyderabad 16. Grant Medical Foundation Ruby Hall Clinic, Pune 17.Sanjeevani Children's hospital, Aurangabad 18. Government Medical College, Chandrapur

Note that full information on the approval of the study protocol must also be provided in the manuscript.

Field-specific reporting

Please select the one below that is the best fit for your research. If you are not sure, read the appropriate sections before making your selection.

☒ Life sciences ☐ Behavioural & social sciences ☐ Ecological, evolutionary & environmental sciences

For a reference copy of the document with all sections, see nature.com/documents/nr-reporting-summary-flat.pdf

Life sciences study design

All studies must disclose on these points even when the disclosure is negative.

Sample size	The sample size was chosen in an iterative, trial-and-error fashion to give the desired power of at least 90%. First, the sample size for LTL consistency was derived and then adjusted with respect to NI for 2:1 allocation ratio (DTwP-HepB-IPV-Hib vaccine: Comparator vaccines).
Data exclusions	The PP included subjects from FAS population who received all study vaccines as per the assigned vaccine group and had pre- and post-dose immunogenicity measurement(s) with no major protocol deviations that were determined to potentially interfere with immune response to the study vaccine. This population served as the primary analysis population for the immunogenicity objectives.
Replication	The findings cannot be replicated.
Randomization	The computer-generated randomisation list (randomisation schedule) for vaccine assignment was generated before the start of the study and randomisation was done through IWRS. At Screening Visit (Visit 1), all eligible subjects were given a randomisation number that assigned them to HEXASIIL™ or SIIPL Pentavac SD + Poliovac vaccine groups. Randomisation was sequential in the order of enrolment and eligibility confirmation. Once a randomisation number was assigned, it was not re-assigned.
Blinding	This was an open-label study. The bioanalytical laboratory performing assay analysis was kept blinded for the treatment administered.

Reporting for specific materials, systems and methods

We require information from authors about some types of materials, experimental systems and methods used in many studies. Here, indicate whether each material, system or method listed is relevant to your study. If you are not sure if a list item applies to your research, read the appropriate section before selecting a response.

Materials & experimental systems

n/a	Involved in the study	
☐	☒	Antibodies
☒	☐	Eukaryotic cell lines
☒	☐	Palaeontology and archaeology
☒	☐	Animals and other organisms
☐	☒	Clinical data
☒	☐	Dual use research of concern
☒	☐	Plants

Methods

n/a	Involved in the study	
☒	☐	ChIP-seq
☒	☐	Flow cytometry
☒	☐	MRI-based neuroimaging

Antibodies

Antibodies used

Antibodies testing against diphtheria, tetanus and pertussis was performed using commercial CE certified kits (RE56191 for diphtheria, RE56901 for tetanus and RE56141 for pertussis; IBL International GmbH, Germany). The VaccZymeTM (MK016.U; Binding Site Group Ltd., United Kingdom) and ARCHITECT Anti-HBs (Abbott Laboratories, Ireland) commercial kits were used for antibodies against Hib and Hepatitis B, respectively.

Validation

Antibodies testing against diphtheria, tetanus and pertussis was performed using commercial CE certified kits (RE56191 for diphtheria, RE56901 for tetanus and RE56141 for pertussis; IBL International GmbH, Germany). The VaccZymeTM (MK016.U; Binding Site Group Ltd., United Kingdom) and ARCHITECT Anti-HBs (Abbott Laboratories, Ireland) commercial kits were used for antibodies against Hib and Hepatitis B, respectively. The kits were validated using international reference standards as per ICH, US FDA and EMEA guidance on bioanalytical methods.

Clinical data

Policy information about clinical studies
All manuscripts should comply with the ICMJE guidelines for publication of clinical research and a completed CONSORT checklist must be included with all submissions.

Clinical trial registration

CTRI/2019/11/022052

Study protocol

Study protocol will be made available from the corresponding author upon reasonable request.

Data collection

Study was carried out at 18 hospitals in India. Data collection was done form February 2020 to March, 2021.

Outcomes

Primary outcome - For each antigen/serotype, non inferiority was shown if a two-sided 95% confidence interval for the absolute difference in response proportions in terms of seroprotection/seroconversion [proportion of responders with DTwP-HepB-IPV-Hib vaccine minus proportion with Comparator (DTwP-HepB-Hib + IPV)] has lower limit > -10%.
Secondary outcome - Lot to lot consistency would be demonstrated if the lower and upper limits of the two-sided 95% CIs for GMC/GMT ratio between each pair among the 3 lots of DTwP-HepB-IPV-Hib was within the pre-defined equivalence limits of [0.5 to 2] for all antigens/serotypes.

Plants

Seed stocks

Report on the source of all seed stocks or other plant material used. If applicable, state the seed stock centre and catalogue number. If plant specimens were collected from the field, describe the collection location, date and sampling procedures.

Novel plant genotypes

Describe the methods by which all novel plant genotypes were produced. This includes those generated by transgenic approaches, gene editing, chemical/radiation-based mutagenesis and hybridization. For transgenic lines, describe the transformation method, the number of independent lines analyzed and the generation upon which experiments were performed. For gene-edited lines, describe the editor used, the endogenous sequence targeted for editing, the targeting guide RNA sequence (if applicable) and how the editor was applied.

Authentication

Describe any authentication procedures for each seed stock used or novel genotype generated. Describe any experiments used to assess the effect of a mutation and, where applicable, how potential secondary effects (e.g. second site T-DNA insertions, mosiacism, off-target gene editing) were examined.
